# Cancer and aging: A call to action

**DOI:** 10.1002/aac2.12055

**Published:** 2022-07-12

**Authors:** Dejana Braithwaite, Stephen Anton, Supriya Mohile, James DeGregori, Nancy Gillis, Daohong Zhou, Shirley Bloodworth, Marco Pahor, Jonathan Licht

**Affiliations:** 1 Departments of Surgery and Epidemiology, University of Florida, Gainesville, Florida, USA; 2 University of Florida Health Cancer Center, University of Florida, Gainesville, Florida, USA; 3 Institute on Aging, University of Florida, Gainesville, Florida, USA; 4 Department of Medicine, University of Rochester Medical Center, Rochester, New York, USA; 5 Department of Biochemistry and Molecular Genetics, University of Colorado, Aurora, Colorado, USA; 6 Department of Cancer Epidemiology and Malignant Hematology, Moffitt Cancer Center, Tampa, Florida, USA; 7 Department of Biochemistry and Structural Biology, University of Texas Health Sciences Center at San Antonio, San Antonio, Texas, USA

**Keywords:** aging, cancer, transdisciplinary science

## Abstract

**Background:**

The intersection of cancer and aging is an emerging public health challenge in developed countries because of the aging and expansion of the population.

**Aims:**

We convened a panel of experts to share their insights on this topic at the inaugural University of Florida Health Cancer Center’s (UFHCC’s) Cancer and Aging Symposium, which was held virtually in February 2022.

**Methods:**

We featured presentations from four leading scientists, whose research spans multiple disciplines including basic science, translational research, geriatric oncology, and population science.

**Results:**

Each speaker offered their unique perspective and insight on the intersection between cancer and aging and discussed their current and ongoing research in this field. In addition to this panel of experts, scientists from the National Institutes of Health and the National Cancer Institute, as well as a UFHCC-affiliated citizen scientist, shared their perspectives on strategies to move the field forward. Some of the key open questions and opportunities for future research offered by these presenters in aging and cancer include but are not limited to infusing health disparities research into the field of cancer and aging, assessing the value of geriatric assessment in identifying early vulnerabilities that may affect response to emerging cancer therapies in older patients, and assessing biological age and other biomarkers (e.g., clonal hematopoiesis) in relation to clinical endpoints and the development of primary, secondary, and tertiary cancer prevention interventions.

**Conclusion:**

Research is needed to accelerate knowledge regarding the dynamic interplay of cancer and aging and optimize care in diverse older adults to achieve equity in cancer outcomes.

## 1 | OPENING REMARKS

**FIGURE 1:**
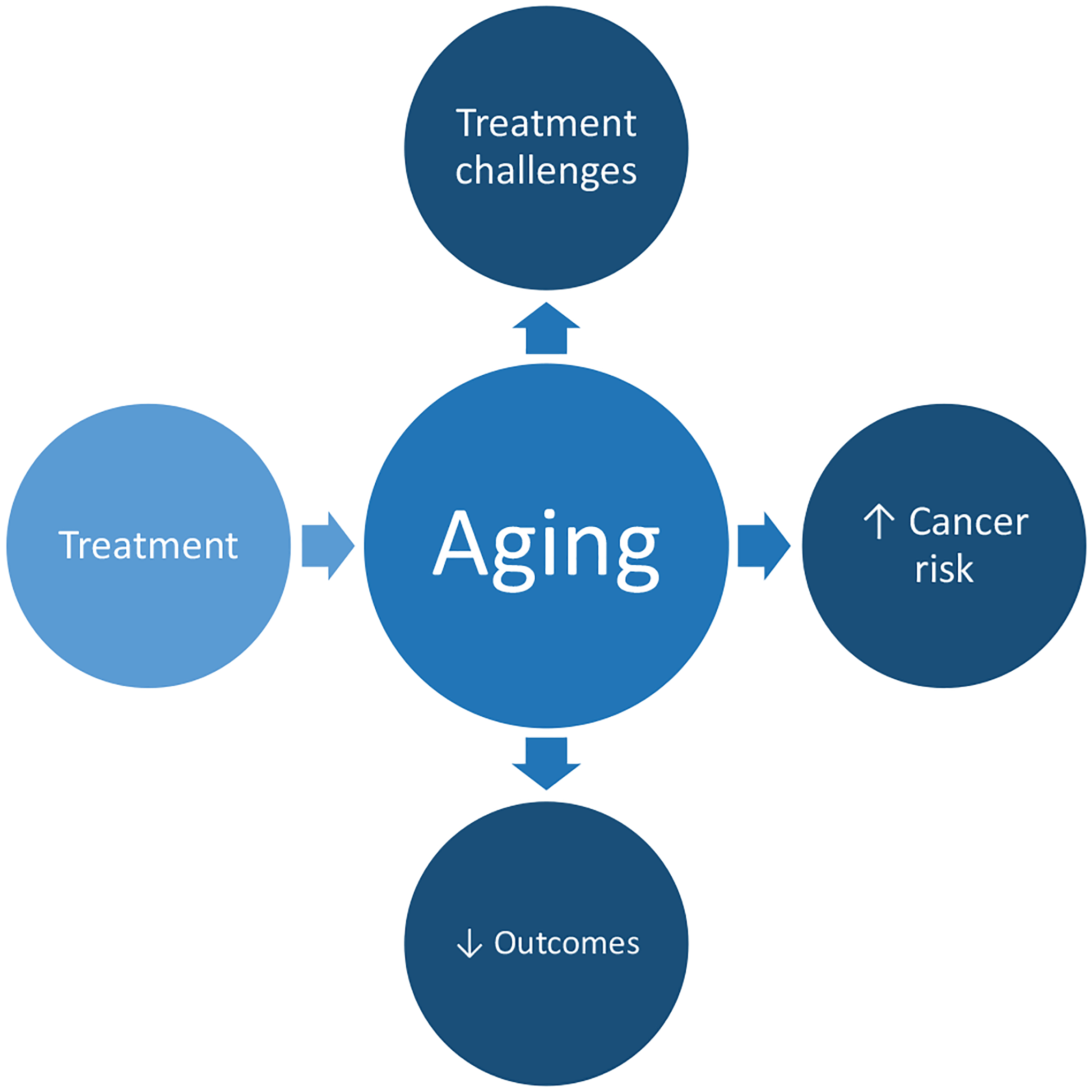
Understanding the interplay between cancer and aging

Rapid population aging, increasing life expectancy, and the rising incidence of cancer with age have led to a significant increase in the incidence of cancer among older populations. Due to these demographic and epidemiological trends,^1^ there is a need to optimize care in diverse older adults to achieve equity in cancer outcomes.^2^ Current estimates suggest that only 24% (i.e., less than a quarter) of participants in trials registered with the US Food and Drug Administration are ≥70 years,^3–6^ and <10% of patients in this age group are enrolled in National Cancer Institute (NCI)-sponsored clinical trials.^7–14^ This poses a clinical and public health conundrum as we must extrapolate data on cancer therapeutics from clinical trials conducted in younger and healthier patients to their older counterparts.^6,15^ Furthermore, older adults who participate in cancer trials tend to have less comorbid conditions^16^ and higher functioning than older patients in real-world settings.^9,13,14,17^ Consequently, there are far-reaching disparities in cancer outcomes between older and younger patients.^18–31^ Thus, more research is needed to examine the unique challenges faced by older adults, such as the impact of age-related deficits and declines in physical and cognitive functioning and the impact of biological age on treatment tolerance and health outcomes. Most common cancers, including lung, colorectal, prostate, and breast malignancies, are diseases of aging. The links between cancer and aging can be seen in the dynamic interplay between the hallmarks of cancer and the hallmarks of aging (Figure 1).^32^ It is now well established that aging and oncogenesis share several mechanisms, including the role of genomic instability, telomere attrition, epigenetic changes, loss of proteostasis, decreased nutrient sensing, and altered metabolism, but also cellular senescence and stem cell function.^33^

As clinical practice increasingly serves growing populations with late-life cancers,^34^ it becomes imperative to apply measures of biological age at the point of care to personalize cancer therapy, develop tailored survivorship care plans, and refine cancer prevention interventions.^2,35^ One of the key challenges for research on cancer and aging is to identify measures or biomarkers of aging that are feasible to implement in clinical care. Epidemiological evidence from longitudinal assessments of biological age in cancer survivors is an important next step to assess and validate promising markers so that evidence-based recommendations can be made to cancer survivors receiving care. We convened a panel of experts to discuss a wide range of topics, as well as National Institutes of Health (NIH) members and citizen scientists to share their views. The research presented spanned multiple disciplines including basic science, clinical research in geriatric oncology, and population science. A summary of each of these presentations follows, along with some of the key open questions and opportunities for future research offered by these presenters in aging and cancer.

### 1.1 | Aging, somatic evolution, and cancer—the inexorable link

Dr. DeGregori presented current research focused on identifying factors that can uncover the mysteries of why cancer is a disease of aging. He described the importance of framing our understanding for why cancer is predominantly a disease of old age through the lens of Life History Theory—the theory describing the evolution of very different patterns of maturation, growth, reproduction, and potential lifespans in different animals. Essentially, researchers need to consider how natural selection has invested in tissue maintenance through years of likely reproductive success, and how the waning of these mechanisms leads to our decline in later years (“aging”). His model of adaptive oncogenesis describes how the alterations in tissue structure and function in older ages create opportunities for selection of new oncogenic phenotypes that are adaptive in this aged tissue landscape.^36^ The two central tenets of this theory are that (1) having high stem cell fitness and healthy tissues actively opposes somatic evolution and thus promotes the status quo, and (2) aging and damage alter the adaptive landscape. Notably, maintaining tissue health in youth not only prevents cancers, but also other diseases associated with older age. Our overall physiological decline with aging (often accelerated by lifestyle choices) promotes not only cancers but other diseases of aging. Dr. DeGregori provided support for this new understanding of cancer genesis, showing how aging can promote selection of oncogenic events that lead to leukemias.^37^ Still, there is some hope as he further showed how restoring more youthful parameters (such as by reducing inflammation) can limit aging-associated oncogenesis. He also presented data showing how inflammation can be sufficient to promote selection for oncogenic events, such as the inactivation of CEBPA (CCAAT enhancer binding protein alpha).^38^ Finally, he presented studies showing how our bodies become riddles with clones driven by oncogenic mutations as we age.^39^

Research has clearly shown that aging is associated with many diseases, from cancers to cardiovascular disease to neurodegeneration to infections.^40^ Similarly, lifestyle choices and exposures (smoking, obesity, lack of exercise, etc.) contribute to the increased risk of many diseases including cancers. A major question is how these different risks are linked—are there changes in our bodies, such as inflammation and epigenetic modifications that underlie these common consequences of aging and lifestyles? And if so, are there interventions that mitigate multiple risks simultaneously? Strategies that center on promoting health behaviors are poised to reduce cancer burden, including optimizing energy balance, eliminating tobacco exposure, reducing alcohol consumption, and increasing vaccine uptake. Optimizing our understanding of mechanisms by which health behaviors and exposures to carcinogens influence physiological and cellular phenotypes that drive the risk of cancer, and chronic disease is critical for optimizing strategies to prevent and treat cancer.^32^ For example, the discoveries of epigenetic clocks and epigenetic drift present such an opportunity,^41^ as studies have shown that DNA methylation changes are commonly driven by changes in the human microbiome, lifestyle factors such as smoking, and exposure to environmental pollutants. Epigenetic modification patterns have also been shown to be associated with exposure to carcinogens such as viruses^42^ and radiation,^43^ but the role of DNA methylation patterns in cancer outcomes is not well-established. Another opportunity^44^ is seen in the discoveries of expanding clones driven by oncogenic mutations in our bodies, as studies have shown how behaviors such as smoking can influence these clonal expansions. Most clearly, studies of clonal hematopoiesis (CH) have demonstrated that the size and identified mutations for these clones can substantially impact disease risk—for not only leukemias, but cancers in general, cardiovascular disease, other diseases and overall mortality.^45^ A key challenge for future research will be to assess the value of such assays (like analyses of blood for CH) in terms of early detection and therapeutic response—who should be offered such tests, and what are the costs and benefits? A more holistic approach to understanding risk across diseases should lead to better methods to assess such risk, develop preventive strategies, enable earlier detection of cancer, and develop better therapies.

### 1.2 | CH and biological aging as predictors of risk for cancer patients

Dr. Gillis presented research focused on CH as a biomarker of aging and predictive marker for patients with cancer. As we age, we accumulate somatic mutations in our hematopoietic stem cells. While most of the mutations are unlikely to induce significant adverse effects, there is a possibility that a stem cell will acquire a mutation that drives clonal expansion. This population of stem cells harboring the same somatic mutation defines CH. While CH is typically considered an event of aging, recent modeling studies suggest that perhaps CH mutations arise early on in life or in utero and expand to detectable levels with aging and other undefined precipitating events.^46^ Of individuals with CH, approximately 1%–5% per year will be diagnosed with a hematologic malignancy. Interestingly, CH is also more prevalent in individuals with solid cancers than those without, across all ages.

Cancer patients with CH have worse outcomes when compared to matched counterparts without CH.^47^ Dr. Gillis presented research aimed at addressing why this is the case. In a nested case-control study, Dr. Gillis and colleagues found that patients who developed secondary therapy-related myeloid neoplasms (t-MNs) were significantly more likely to have CH at the time of initial cancer diagnosis when compared to heavily matched cancer patients who did not develop t-MNs.^48^ Importantly, this study found that the majority of t-MN mutations were present at the time of initial cancer diagnosis and persisted or expanded after treatment. This refuted the conventional hypothesis of the t-MN etiology, which was that cancer treatment induced pathogenic t-MN driver mutations. Therefore, CH may represent a novel predictive biomarker, which can be translated to clinical decision-making to decrease risk for t-MNs. Dr. Gillis also presented evidence that CH mutations can be detected as incidental findings on conventional clinical next-generation sequencing of solid tumor biopsies.^49^ Collectively, this work contributed to the first-ever consensus management recommendations for CH detected in patients with solid tumors.^50^

A related research theme focuses on accelerated biological aging in patients with both HIV and cancer. Dr. Gillis presented preliminary data suggesting that biomarkers of aging, including CH and DNA methylation biological clocks, appear more frequently in patients with HIV and cancer compared to similar patients without concurrent HIV infection. Also, given the increased cancer mortality among patients with HIV, this research seeks to identify if accelerated biological aging in patients with HIV and cancer leads to adverse patient outcomes such as tumor recurrence and poor prognosis. A key clinical challenge being addressed by this work is to identify targetable factors that drive poor outcomes for biologically aged cancer patients. Once clearly defined implications are identified, intervention strategies can be implemented to improve outcomes for this growing population of patients.

### 1.3 | Improving care delivery and outcomes for older patients with advanced cancer and their caregivers

Dr. Mohile co-leads the Cancer and Aging Research Group (CARG), a national collaborative network of geriatric oncology investigators, which aims to accelerate high-quality research at the cancer and aging interface and disseminate the findings of such research to the general community.^51^ CARG meets monthly, includes investigators from all over the US, and provides opportunities for junior researchers to showcase their works and receive mentorship.^51^ The mission of Dr. Mohile’s research program is to improve outcomes among older populations with cancer through applications of comprehensive geriatric assessment tools that facilitate early identification of significant adverse effects of the malignancy and its treatment due to age-related medical, cognitive, functional, nutritional, and psychosocial factors.

A recently completed study includes a Patient-Centered Outcomes Research Institute—funded multicentered clinical trial to evaluate whether the geriatric assessment can improve communication about age-related concerns of older patients with advanced care and their caregivers.^52^ Studies have found that not only can the geriatric assessment be used to help improve patient-provider communication, but it can also help identify challenges that their caregivers are facing. Other studies completed recently, funded by the NIH, sought to evaluate whether care management recommendations based on geriatric assessment results could reduce chemotherapy toxicity. A large cluster randomized study found that a geriatric assessment intervention lowered the risk of developing serious chemotherapy toxicity in older adults with aging-related conditions receiving treatment for advanced cancer.^53^ A key challenge for future research will be to assess the value of integrating measures of biological age at the point of care to personalize cancer therapy and mitigate toxicity.

Dr. Mohile discussed some of the clinical challenges and opportunities regarding care of older patients receiving immunotherapy, which has significantly changed the landscape of caring for these patients. She noted that older patients with cancer benefit from immune checkpoint inhibitors but have higher rates of adverse events and therapy discontinuation with increasing age.^54^ Dr. DeGregori added that adaptive immunity decreases with age, which is due to an increase in proteins such as PD-L1 and PD-1. This means that checkpoint inhibitors may still work effectively in these patients, as the therapies are undoing the suppression that is observed with age.

### 1.4 | Senescent cells are novel targets for aging and cancer

Dr. Zhou’s research focuses on the role of senescent cells in aging and cancer and the development of senolytics, novel agents that selectively target senescent cells. Senescence is one mechanism whereby tumor cells avoid the direct cytotoxic impact of cancer therapy, which allows extended survival in a dormant state, with the possibility of recovering capacity for self-renewal and contributing to cancer recurrence.^55^ Senescence can be a consequence of treatment with inhibitors of cyclin-dependent kinases 4 & 6 (CDK4/6),^56,57^ Polo and Aurora kinases,^57,58^ histone deacetylases, and other epigenetic modifiers.^59^ Moreover, cells can join the cell cycle following a prolonged senescence arrest to produce progeny characterized by chromosomal instability or a cancer stem cell-like phenotype,^60^ thus providing a survival advantage. Discerning “irreversible senescence arrest” versus “senescence-like arrest”^61^ is important because the cells rejoining the cell cycle following senescence-like arrest may contribute to treatment failure.

Specifically, senolytics may delay age-related pathologies, treat age-related diseases, extend an individual’s health span and lifespan, increase tumor responses to treatment, reduce tumor relapse and metastasis, and prevent or reverse side-effects of cancer treatment.^62^ Studies of a senolytic agent, navitoclax (ABT263) (a B-cell lymphoma-extra-large [BCL-XL] and B-cell lymphoma 2 [BCL-2] dual inhibitor), revealed that they can reverse side effects of radiation exposure such as radiation-induced pulmonary fibrosis and bone marrow suppression.^63,64^ Similarly, studies found that clearance of senescent cells with ABT263 can also reduce side effects of chemotherapy such as chemotherapy-induced cardiotoxicity, fatigue and myelosuppression^65,66^ and even partially reverse cisplatin-induced peripheral neuropathy. Furthermore, in combination with the cytotoxic chemotherapy agent doxorubicin, ABT263 reduced tumor size and metastasis in a mouse breast cancer model.^65^ Unfortunately, ABT263 causes on-target, dose-limiting platelet toxicity, which limits its clinical application as a therapeutic. This prompts the discussion of how proteolysis-targeting chimera (PROTAC) technology may be used to reduce platelet toxicity of ABT263 while improving its senolytic activity. Zhou developed a PROTAC that induces cell/tissue-specific degradation of BCL-XL by recruiting an E3 ubiquitin ligase differentially expressed in senescent cells but not in platelets to prevent unwanted toxicity.^67,68^ A related challenge is to determine whether this technology may be applicable to other toxic senolytics.

## 2 | CONCLUSION

Following the presentations from our speakers, we were fortunate to have the perspective of a citizen scientist, as well as three representatives from the NCI. Two themes emerged during this discussion. First, it was noted that there is a need for researchers to focus more on enhancing meaning and quality of life in older cancer survivors in particular. Second, there was a consensus on the importance of including citizen scientists in the design and implementation of interventions at the intersection of cancer and aging. Following this discussion, the three representatives from the NCI indicated that advancing research on cancer and aging is a major goal of the NCI’s strategic plan, and a clinical and public health priority. They also shared current funding opportunities relevant to cancer and aging, which can be accessed on NCI’s aging and cancer webpage at: https://cancercontrol.cancer.gov/brp/bbpsb/aging-and-cancer.

### 2.1 | Future implications and challenges

The presentations provided by our experts highlight the complexity involved in the field of cancer and aging. Despite important advances, cancer and aging remains a nascent field with little formal infrastructure to bring together scientists and advocates to move the field forward. The integration of basic, clinical, and population sciences is necessary to tackle the relationship between cancer and aging, as well as cancer and the emergence of other diseases. Although we have seen from our speakers the tremendous progress in the realm of cancer and aging, there is still a significant need to address the unique challenges faced by a growing demographic of racially and ethnically diverse older adults. One such challenge is over-coming the limited efficacy of immunotherapies in older cancer patients that results from multiple factors, including immunosenescence—immunological decline characterized by an increase in memory T cells and decreased peripheral blood naïve cells.^69^ Although immunotherapies are revolutionizing treatment landscape, the efficacy of immunotherapy appears to decrease with age,^70^ and older adults are more likely to experience immunotherapy-related adverse events,^71^ which often result in early discontinuation of treatment.^72^ Clearly, it is imperative that we elucidate the dynamic interplay of aging, cancer, and immunosenescence. Another significant challenge for future research is concerned with infusing health disparities research into the field of cancer and aging. President Biden’s recently reignited Cancer Moonshot program aims to reduce cancer deaths and improve the quality of life of cancer survivors and their families, but to reach these goals, the relationship between cancer and aging, and attendant disparities, must remain at the forefront of research initiatives.^73^

Examples of important areas in need of further research include (but are not limited to):

1. Investigation of aging trajectories associated with specific cancer treatments and identification of cancer survivors at risk for an “accelerated aging” phenotype;
2. Examination of the effects of specific cancer treatments on aging biology, such as at signaling and gene expression levels, that may alter aging trajectories or aging outcomes;
3. Development and/or validation of tools, technologies, measures, or techniques for the identification of late-emerging effects and aging phenotypes;
4. Development and/or testing of interventions designed to prevent, mitigate, or reverse the adverse aging-related effects of cancer and cancer treatments;
5. Development and/or testing of interventions focused on models and processes of care delivery to intervene at the intersection of cancer treatment and aging;

All of these areas need to ensure meaningful inclusion of racially and ethnically diverse populations.
